# Rapidly Progressive Medial Calcific Sclerosis in a Dialysis Patient

**DOI:** 10.3390/diagnostics16111609

**Published:** 2026-05-25

**Authors:** Robiel Habtemariam Tesfu, Guillaume Fahrni

**Affiliations:** Department of Diagnostic and Interventional Radiology, Lausanne University Hospital and University of Lausanne, 1011 Lausanne, Switzerland

**Keywords:** Mönckeberg sclerosis, medial calcific sclerosis, dialysis, abdominal aorta, computed tomography

## Abstract

Medial calcific sclerosis (Mönckeberg sclerosis) is a common but often underrecognized complication in patients with end-stage renal disease (ESRD). Unlike atherosclerosis, it is characterized by circumferential, continuous calcification of the arterial media. We present the case of a 41-year-old man on dialysis who exhibited a strikingly rapid progression of diffuse medial calcifications of the abdominal aorta and its major branches within a 10-month period. This case emphasizes the clinical importance of distinguishing this pattern on computed tomography (CT) to identify patients at heightened cardiovascular risk.

**Figure 1:**
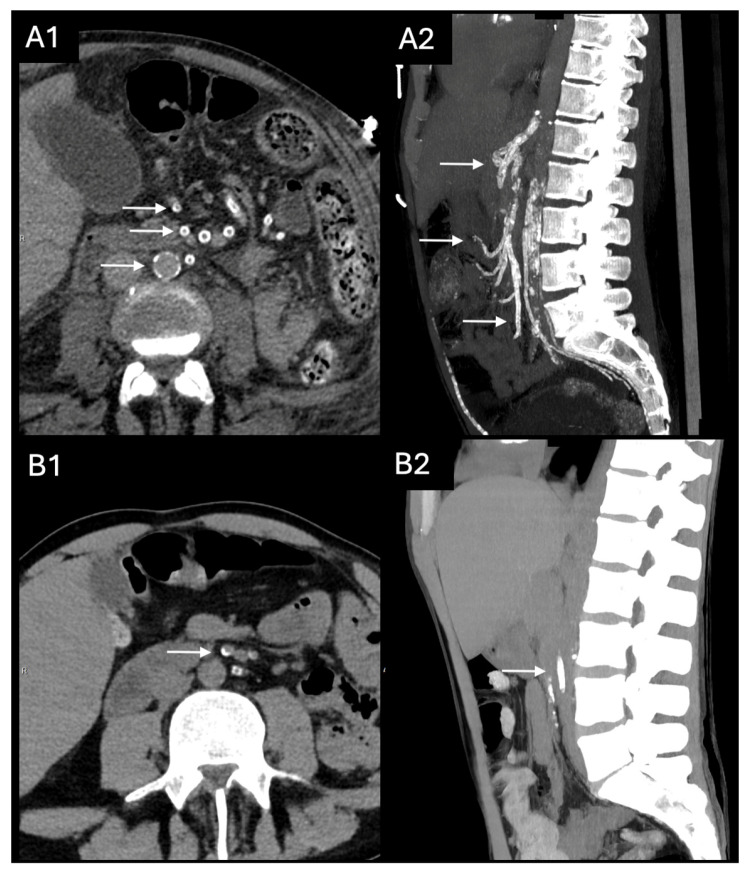
Abdominal CT images from the current examination (**A1**,**A2**) and 10 months prior (**B1**,**B2**). (**A1**) Axial multiplanar reformation and (**A2**) sagittal maximum intensity projection (MIP) views showing extensive, diffuse, circumferential calcification of the abdominal aorta and its major branches (white arrows). (**B1**) Axial view and (**B2**) sagittal MIP view from 10 months prior, showing only minimal scattered calcifications in the visceral branches. A 41-year-old man with end-stage renal disease (ESRD, Stage G5) on maintenance intermittent hemodialysis via a tunneled catheter was admitted for multidrug-resistant *Pseudomonas aeruginosa* pneumonia. His clinical history was notable for profound therapeutic non-compliance, presenting with untreated hypertension and untreated hypercholesterolemia. Contrast-enhanced thoracoabdominal CT revealed extensive, diffuse, circumferential calcification of the abdominal aorta and its major branches. A retrospective review of a CT performed only 10 months earlier showed minimal calcifications, highlighting a strikingly rapid progression likely driven by poor therapeutic compliance and unmanaged cardiovascular risk factors. The patient unfortunately passed away shortly after these examinations due to a severe respiratory infection. The imaging pattern observed, characterized by regular, circumferential, and continuous calcifications, is typical of medial (Mönckeberg) calcific sclerosis. Despite the patient’s hypercholesterolemia, this diffuse and rapidly progressive pattern allows differentiation from atherosclerotic vascular calcification, which usually presents with irregular, eccentric, intimal-based deposits [1,2]. In addition, although less frequently, similar calcification patterns may be encountered in other vascular conditions or in patients with overlapping clinical presentations, particularly in end-stage renal disease, potentially leading to unnecessary additional imaging or invasive diagnostic procedures if misinterpreted. Therefore, correct radiological recognition of this entity on CT is essential for accurate diagnosis and appropriate clinical management. Medial calcification is common in chronic kidney disease associated with calcium–phosphate imbalance [3]. At the molecular level, hyperphosphatemia is thought to promote medial calcification by driving vascular smooth muscle cells toward an osteogenic phenotype rather than simple passive mineral deposition. Experimental and clinicopathologic studies suggest that phosphate retention in uremia activates pathways linked to Pit-1 signaling, oxidative stress, and osteochondrogenic reprogramming, with early upregulation of Runx2 and osteopontin and loss of smooth muscle markers [4]. In human Mönckeberg lesions, medial calcification appears to be an active process orchestrated by phenotypically modified VSMCs, supporting the concept that chronic phosphate burden acts as a major amplifier of CKD-related medial arterial calcification [5]. In this uremic terrain, the severe septic episode may have acted as an acute inflammatory catalyst, where systemic cytokines (such as TNF-α and IL-6) further amplified VSMC osteogenic transdifferentiation, accelerating the progression toward a diffuse pattern [6]. While often asymptomatic, Mönckeberg’s arteriosclerosis increases cardiovascular risk due to arterial stiffening. In the dialysis setting, this severe loss of arterial compliance and increased pulse pressure significantly compromise vascular access planning, impairing arteriovenous fistula maturation and long-term patency, or causing severe intradialytic ischemic pain that may mandate access closure [7]. The most frequent clinical manifestation is the development of peripheral ulcers, particularly in the distal lower limbs, which can occasionally progress to critical ischemia and require amputation [8]. Currently, no pharmacologic treatment halts progression, leaving revascularization as the only option for symptomatic patients. From a preventive standpoint, progression of medial calcific sclerosis in chronic kidney disease is primarily driven by CKD-mineral and bone disorder, particularly hyperphosphatemia, hypercalcemia, and secondary hyperparathyroidism. Therefore, mitigation strategies rely on strict control of serum phosphate through dietary restrictions, optimized dialysis, and phosphate binders, together with management of classical cardiovascular risk factors such as hypertension and dyslipidemia, even though no therapy has been proven to reverse established vascular calcification [9]. The characteristic CT findings include linear vascular calcifications in the medial layer of arteries, which are nonocclusive and unrelated to atherosclerosis. Common sites of involvement include the iliac arteries (often affected earliest), renal arteries, and branches of the celiac and superior mesenteric arteries, including the hepatic and splenic arteries [10].

## Data Availability

The original contributions presented in this study are included in the article. Further inquiries can be directed to the corresponding author.

## Abbreviations

The following abbreviations are used in this manuscript:

CKD	Chronic Kidney Disease
CT	Computed Tomography
MIP	Maximum Intensity Projection
ESRD	End-Stage Renal Disease
VSMC	Vascular Smooth Muscle Cells
